# Trends in Incidence and Mortality of Primary Cutaneous Melanoma in Serbia Between 2008 and 2022

**DOI:** 10.3390/medicina62040741

**Published:** 2026-04-13

**Authors:** Zorana Babic, Snezana Radovanovic, Sanja Kocic, Svetlana Radevic, Katarina Janicijevic, Sanja Ilic, Jovana Radovanovic Selakovic, Vladimir Vukomanovic, Milos Stepovic, Olgica Mihaljevic, Biljana Seha, Jelena Milin-Lazovic, Marko Petrovic, Marina Kostic, Ognjen Djordjevic, Ivana Simic Vukomanovic

**Affiliations:** 1 Faculty of Medical Sciences, University of Kragujevac, 34000 Kragujevac, Serbia; zoranakovinic@gmail.com; 2 Department of Social Medicine, Faculty of Medical Sciences, University of Kragujevac, 34000 Kragujevac, Serbia; jovanarad@yahoo.com (S.R.); kocicsanja@yahoo.com (S.K.); kaja.andreja@yahoo.com (K.J.); sanjailic84@yahoo.com (S.I.); drivanasimic@gmail.com (I.S.V.); 3 Institute of Public Health Kragujevac, 34000 Kragujevac, Serbia; ognjendjordjevic763@gmail.com; 4 Department of Epidemiology, Faculty of Medical Sciences, University of Kragujevac, 34000 Kragujevac, Serbia; radovanovicjovanaaa@gmail.com; 5 Department of Nuclear Medicine, Faculty of Medical Sciences, University of Kragujevac, 34000 Kragujevac, Serbia; vukomanovic@gmail.com; 6 Department of Anatomy, Faculty of Medical Sciences, University of Kragujevac, 34000 Kragujevac, Serbia; stepovicmilos@yahoo.com; 7 Department of Pathophysiology, Faculty of Medical Sciences, University of Kragujevac, 34000 Kragujevac, Serbia; vrndic07@yahoo.com; 8 Center for Radiology, Department of Stereotactic Radiosurgery, University Clinical Center of Serbia, 11000 Belgrade, Serbia; biljana.seha@yahoo.com; 9 Institute for Medical Statistics and Informatics, Faculty of Medicine, University of Belgrade, 11000 Belgrade, Serbia; 10 Department of Surgery, Faculty of Medical Sciences, University of Kragujevac, 34000 Kragujevac, Serbia; markopetrovickg@yahoo.com; 11 Department of Pharmacology and Toxicology, Faculty of Medical Sciences, University of Kragujevac, 34000 Kragujevac, Serbia; marrina2006kg@yahoo.com

**Keywords:** cutaneous melanoma, incidence, mortality, epidemiology, trends

## Abstract

*Background and Objectives:* Melanoma is one of the most aggressive malignancies in humans and is associated with an unfavorable prognosis due to its high metastatic potential. In recent decades, increasing incidence and mortality rates have been reported worldwide among Caucasian populations. This study aimed to analyze melanoma incidence and mortality trends in the Republic of Serbia from 2008 to 2022, with a special regard to sex- and age-specific differences. *Materials and Methods:* A descriptive epidemiological study was conducted using data on all cases of primary cutaneous melanoma (ICD-10 code C43) reported to the Serbian Cancer Registry between 2008 and 2022. Data on newly diagnosed cases and melanoma-related deaths were analyzed by sex and 5-year age groups. Crude rates were calculated per 100,000 populations, whereas age-standardized rates were obtained using direct standardization. Temporal trends were evaluated using the Joinpoint regression analysis. *Results:* During the study period, 9600 new melanoma cases and 3882 melanoma-related deaths were reported in Serbia. Both incidence and mortality were higher in men (52.5% and 60.1%, respectively). Age-standardized incidence rates remained relatively stable in both sexes, with a slight increase in men (AAPC = 0.9%; 95% CI: −0.5 to 2.3) and a stable trend in women (AAPC = −0.1%; 95% CI: −1.5 to 1.3). Age-standardized mortality rates showed a mild, statistically insignificant decline overall (AAPC = −0.6% in men and −0.9% in women). The calculated mortality-to-incidence ratio (MIR) shows consistently higher values in men compared to women. A significant increase in incidence was observed among men aged 70–74 years (AAPC = 3.1%; *p* = 0.011), while mortality significantly decreased in women aged 40–44 years (APC = −7.0%; *p* = 0.017). *Conclusions:* Melanoma incidence in Serbia remained relatively stable between 2008 and 2022, with a modest increase primarily among older men. Mortality trends showed a slight decline, suggesting potential improvements in early detection and treatment outcomes.

## 1. Introduction

Melanoma is a malignant tumor resulting from the uncontrolled proliferation of melanocytes—the pigment-producing cells [1]. The most common form of melanoma is cutaneous, but it can also develop in mucosal surfaces, the uvula tract and leptomeninges.

Melanoma is one of the most aggressive malignancies in the human population, characterized by a high level of metastasizing, and therefore is associated with a relatively unfavorable prognosis. A rise in the incidence rates for melanoma has been noted among Caucasians worldwide for the past few decades, whereas the incidence rates in the darker-skinned populations of Africa and Asia are still at a low level. The annual incidence rate has risen between 3 and 7% in numerous fair-skinned populations prevailing in regions such as Australia and New Zealand, North America, and North Europe [2]. Although it accounts for less than 5% of all skin cancers, it causes approximately 80% of cancer-related deaths in that particular group due to its aggressive nature and capacity for rapid extension through various pathways, including lymphatic and hematogenous spread [1]. Known risk factors contributing to the increasing incidence of melanoma in the Caucasian population include excessive ultraviolet (UV) radiation exposure (from sunlight and tanning beds), fair skin phenotype, genetic predisposition, history of sunburns (especially in childhood), and increased surveillance and early detection practices [2,3].

The number of new cases of melanoma in the future is estimated to increase by 50% by 2040, from 325,000 cases in 2020 to 510,000 cases in 2040. Similarly, the number of melanoma-related deaths is expected to rise by approximately 68%, from 57,000 in 2020 to 96,000 in 2040 [3].

Although the incidence rates are continuing to rise in the majority of European countries, several high-risk countries, including Australia, the USA, Scandinavia, and the United Kingdom, have reported a further stabilization and a declining trend in melanoma incidence rates, mostly in the younger age groups [3,4,5]. A rise in melanoma mortality rates has been reported parallel to the overall increasing incidence rates. However, increasing mortality rates seem to have slowed down or have stabilized in certain regions, and several recent studies even suggest a slight decrease reported in younger age groups [6]. These findings may be attributed to the early disease detection and greater availability of modern life extension treatments.

All over the world, there are significant disparities in the collection and availability of precise epidemiological data, screening implementation and early diagnosis capacities, along with the availability of modern therapies, reflecting the greater differences in the malignant melanoma incidence, mortality and survival trends [7].

Numerous factors play a role in increasing melanoma incidence rates, such as a higher UV exposure, lifestyle changes, a higher level of awareness of early disease detection and more advanced diagnostics [8]. In addition, the registration of cases in the national registries and enhancement of epidemiological data quality contribute to a better understanding of the real-world disease burden.

Melanoma represents an increasing challenge for the public healthcare system in the Republic of Serbia, as well as for the majority of other countries in the region. The previous studies indicate a gradual rise in the melanoma incidence rates, followed by trends in mortality variability [9,10,11,12,13]. However, comprehensive data on trends by age and sex are limited, and there are no systematic analyses covering longer-term periods.

Investigating the changes in the incidence and mortality rates over time, including the sex-specific and age-specific differences, is of paramount importance for a better understanding of melanoma epidemiology and planning preventive measures.

This paper presents an analysis of the incidence and mortality rates for melanoma in the Republic of Serbia in the period from 2008 to 2022, with special regard to the gender-specific and age-specific differences.

## 2. Materials and Methods

### 2.1. Data Sources

Research has been conducted as a descriptive epidemiological study based on all cases of primary cutaneous melanoma (International Classification of Disease, Tenth Revision [ICD-10] code C43), reported to the Serbian Cancer Registry during the period from 2008 to 2022. The study covered the territory of the Republic of Serbia, excluding the Autonomous Province of Kosovo and Metohija as no official data have been available from this region since 1998.

The data on the number of newly diagnosed patients and deaths from skin cancer were obtained by sex and 5-year age groups (i.e., 0–4, 5–9, 10–14, 15–19, 20–24, 25–29, 30–34, 35–39, 40–44, 45–49, 50–54, 55–59, 60–64, 65–69, 70–74 and ≥75 years). The calculation of rates was performed using the data on the number of people based on the results of the population census and official estimates provided by the Statistical Office of the Republic of Serbia. The use of 5-year age groups was selected in accordance with standard cancer epidemiology practice, as it allows for a precise estimation of age-specific rates and facilitates comparability with international cancer registry data. Additionally, this level of stratification enables better identification of age-related patterns in melanoma incidence and mortality.

### 2.2. Statistical Analysis

Crude incidence and mortality rates (CRs) were calculated per 100,000 population, whereas age-standardized rates (ASRs) were calculated using direct standardization with the world standard population by Segi [14].

Incidence and mortality trends were analyzed using the Joinpoint regression analysis, which ensures the identification of points where a statistically significant change in trends occurs. The annual percent change (APC) was computed for each segment between the joinpoints, whereas the average annual percent change (AARC) was computed for the entire observed study period, with a confidence interval of 95% (95% CI). The independent variable was the year, and the dependent variable was the respective rate. The statistical significance is set at the level of 0.05 (*p* < 0.05).

The analyses were performed by sex and five-year age group separately, utilizing sex-disaggregated data from the Serbian Cancer Registry, without using aggregated values. This approach complies with the recommendations of research findings in melanoma epidemiology, taking into account the existing biological and hormonal differences, complete with behavioral variations between men and women.

All calculations were performed using the Joinpoint Regression Program, Version 5.4.0, Statistical Research and Applications Branch, National Cancer Institute [15,16].

## 3. Results

From 2008 to 2022, a total of 9600 new melanoma cases (5040 males, 4560 females) and 3882 deaths from malignant melanoma (2335 males, 1547 females) were reported in Serbia. The number of newly diagnosed cases (52.5%) and deaths (60.1%) was reported to be higher among men. Over the entire observed period, men accounted for the majority of newly diagnosed cases on average, ranging from approximately 48% to 56% annually. The proportion of men among those who died of melanoma ranged from approximately 56% to 63% annually, whereas the proportion of women varied between 37% and 44%. The sex-related structure of newly diagnosed cases and melanoma-caused deaths indicates a stable pattern with a consistently larger proportion of men compared to women over the entire observed period.

The average age-standardized incidence rate (ASIR) was 5.6/100,000 for men and 4.8/100,000 for women. The average age-standardized mortality rate (ASMR) was 2.6/100,000 for men and 1.5/100,000 for women. In general, the ASMR of melanoma was approximately 1.7 higher for males compared to females.

The calculated mortality-to-incidence ratio (MIR), derived from ASIR and ASMR values, shows consistently higher values in men compared to women across all observed years. In men, the MIR ranges from 0.35 to 0.61, while in women, it ranges from 0.22 to 0.40, indicating a generally less favorable cancer survival profile among males. Women demonstrate lower and more stable MIR values, suggesting comparatively better outcomes and potentially more effective early detection or treatment. The observed sex-based disparity in MIR highlights differences in cancer burden and healthcare effectiveness, with men experiencing higher mortality relative to incidence, which may reflect later diagnosis, differences in tumor biology, or variations in access to and utilization of healthcare services.

The number of incidence and death cases, crude rate (CR), ASIR, ASMR, and MIR over the observed period are shown in Table 1.

There were no cases of melanoma registered within the pediatric population aged 0 to 14 years. A greater proportion of newly diagnosed female patients was reported in younger and middle age groups, compared to men, whereas the distribution pattern increased significantly with age. The proportion of men seemed to increase from the age group 50–54 years, and the majority of newly diagnosed patients were registered in older age groups. The majority of cases in both sexes were registered among individuals aged 75+, accounting for almost one-fifth of the total number of newly diagnosed patients. Such a distribution indicates a marked increase in the melanoma burden with advancing age, along with sex-related differences that vary depending on the age group.

Death outcomes were rarely reported at younger ages, whereas the majority of mortality was observed in those over the age of 50. A higher number of death outcomes was reported in men compared with women in the 55–69 age groups, whereas a higher proportion of women who died of melanoma was reported in the oldest (75+) age group. Such a pattern indicates clear age- and sex-related disparities in melanoma mortality rates, with the highest burden observed among advanced age groups (Table 2).

**Figure 1:**
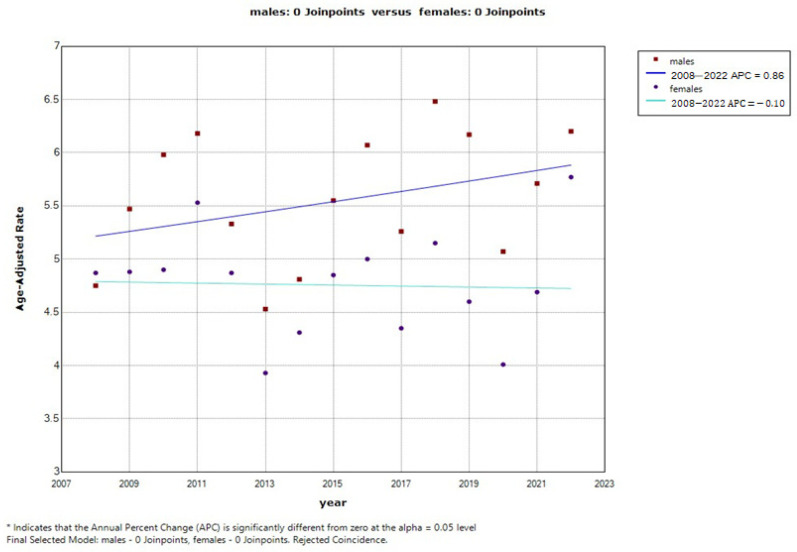
Joinpoint regression analysis of cutaneous melanoma incidence by gender in Serbia from 2008 to 2022.

Both men and women consistently maintained a stable ASIR, with no statistically significant changes observed. A slight increase in the ASIR values was reported for men, from 4.75/100.000 in 2008 to 6.20/100.000 in 2022, with no statistically significant differences in trend change (AAPC = 0.9%; 95% CI: from −0.5 to 2.3; *p* = 0.205), whereas the ASIR values remained stable for women, ranging from 4.87/100.000 in 2008 to 5.77/100.000 in 2022, with a slightly downward trend (AAPC = −0.1%; 95% CI: −1.5–1.3; *p* = 0.885) (Figure 1).

**Figure 2:**
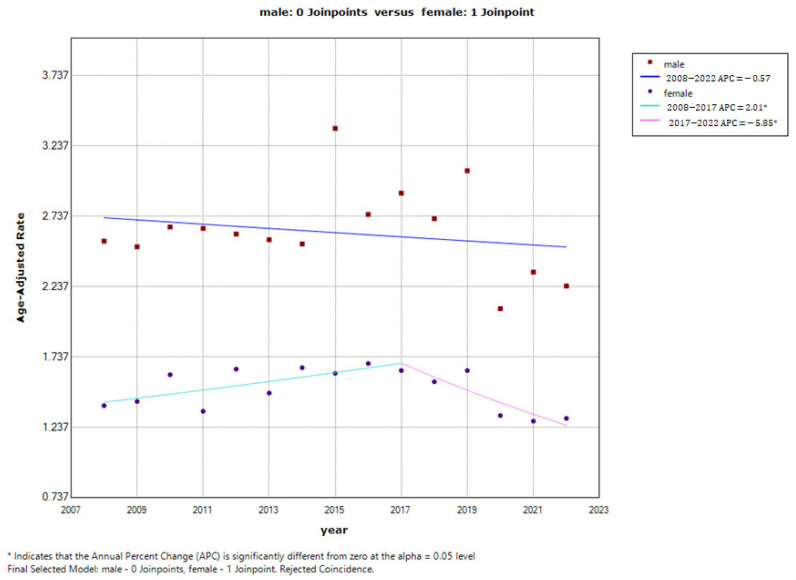
Joinpoint regression analysis of cutaneous melanoma mortality by gender in Serbia from 2008 to 2022.

The ASMR analysis showed a slight decrease in men—from 2.56/100,000 in 2008 to 2.24/100,000 in 2022 (AAPC = −0.6%; 95% CI: −2.1 to 1.0; *p* = 0.441), without statistical significance. One joinpoint was identified in women in 2017, with a statistically significant increase observed in the first segment (2008–2017: APC = 2.0%; 95% CI: 0.0 to 4.0; *p* = 0.046), which was subsequently followed by a considerable decline observed in the second segment (2017–2022: APC = −5.9%; 95% CI: −10.2 to −1.3; *p* = 0.018). The average annual percent change (AAPC) over the entire observed period was −0.6% for men (95% CI: −2.1 to 1.0; *p* = 0.441) and −0.9% for women (95% CI: −2.7 to 1.0; *p* = 0.356), indicating a mild, but statistically insignificant decline in the melanoma mortality rates in the entire population (Figure 2).

The analysis of temporal trends in specific age-standardized incidence rates of melanoma shows that the risk of developing disease significantly increases with age, particularly in older populations. Incidence rates were extremely low in younger age groups (under the age of 40), and they did not show any statistically significant changes over the observed period, 2008–2022. Contrary to these facts, an increasing trend in the incidence rates was observed in the middle-aged and older age groups, which was particularly pronounced in men. A statistically significant increase in melanoma-specific incidence rates was reported in men in the 70–74 age group, with the average annual percent change (AAPC) of 3.1% (95% CI: 0.8% to 5.4%; *p* = 0.011), indicating a significant and persistent increase in the incidence rates over the analyzed period. A considerably milder trend was reported in women of the same age, with no statistically significant changes (with the AAPC of 0.8%; 95% CI: −0.7% to 2.2%; *p* = 0.265). In addition, in other categories (for instance, in the 65–69 age group, and in the 75+ age group), an increase in the incidence rates was observed in both sexes, but those trends failed to reach the statistical significance threshold. In the youngest age groups (younger than 30 years), the incidence rates were consistently low; therefore, no significant variations in trends were observed due to small values. To sum up, the highest increase in incidence rates was reported in older men, whereas melanoma-specific incidence rates in younger adults were relatively stable during the analyzed period (Table 3).

Melanoma-specific mortality rates increase with age, similarly to melanoma incidence rates, and the highest rates were reported in the oldest age groups. However, during the period of 2008–2022, the majority of age categories failed to show a statistically significant trend in mortality changes. Mortality trends remained mostly stagnant in men from all 5-year age groups (without significant growth or decline), even in the advanced age groups, where the absolute risk was considered to be the highest. Men older than 70 years were at the highest risk, with the highest mortality rates, yet this trend remained fairly stable. A statistically significant decline in mortality was reported in women aged 40–44 years (the APC = −7.0%; 95% CI: −12.3 to −1.5; *p* = 0.017). In other female age groups, mortality trends were not statistically significant. The following three trend segments were identified in men older than 75 years: during the period of 2008–2010, an abrupt, but insignificant increase was reported (the APC = 45.9%; 95% CI: −12.1 to 142.1; *p* = 0.121); during the period of 2010–2018, a moderate increase was reported (the APC = 5.1%; 95% CI: −1.8–12.4; *p* = 0.127); whereas a statistically significant decline in mortality rates was observed in the period from 2018 to 2022 (the APC = −16.6%; 95% CI: −28.9–−2.1; *p* = 0.032). In the female 75+ age group, the trend showed two segments in particular: a significant increase during the period of 2008–2019 (the APC = 9.1%; 95% CI: 4.2 to 14.2; *p* = 0.002), followed by an abrupt and statistically significant decline during the period of 2019–2022 (the APC = −36.5%; 95% CI: −54.8 to −10.9; *p* = 0.014). The AAPC value was 3.1% for men in the 75+ age group (95% CI: −4.6 to 11.5; *p* = 0.442), and −2.9% for women (95% CI: −9.6 to 4.3; *p* = 0.421), without statistical significance (Table 4).

On the whole, the oldest age groups carry the largest mortality burden, but without a clear increasing or decreasing trend over the 15-year observation period.

## 4. Discussion

Melanoma epidemiology in Serbia should be interpreted within the broader context of Eastern European countries, where incidence rates are generally lower compared to Western and Northern Europe, but are accompanied by disproportionately higher mortality rates [4,6,9]. This pattern is commonly attributed to later-stage diagnosis, limited access to early detection programs, and differences in healthcare resources. Our findings are consistent with this regional profile, as Serbia demonstrates relatively low incidence rates alongside less favorable mortality outcomes, particularly in men and older age groups.

Our study results demonstrated that men account for slightly more than half of individuals diagnosed with melanoma in Serbia. Incidence rates were higher in men than women, particularly after the age of 50, which aligns with the latest world trends. Data obtained from America, Europe, Australia and New Zealand indicate the higher incidence rates in women approximately until the age of 50, after which they are higher in men. The age at which the incidence rates of melanoma in men surpass the incidence rates in women varies among different population groups; the earliest age at diagnosis is reported in Australia (the 45–49 age group), and the latest age at diagnosis is reported in Denmark (the 65–69 age group) [17]. As regards the advanced age groups, males are at a considerably higher risk—for instance, in the 80+ age group, the incidence of melanoma is three times higher in men than in women of the same age [10]. The prevalence of older age groups in both the incidence and mortality of melanoma is a world-known trend, with higher risks consistently being reported in males in almost all regions [3]. Biological and behavioral differences contribute to such inequality—men are more frequently exposed to UV radiation and are less observant about skin changes [18]. Indeed, numerous studies indicate that the low incidence of melanoma in Serbia and neighboring countries is followed by a high percentage of so-called fatty tumors (increased Breslow thickness) and a significant proportion of younger patients with advanced tumors [4,13]. These results highlight the need for continuous cancer prevention measures and early diagnostics—specifically directed at men and older population groups—to reduce sex inequalities and improve disease outcomes.

The study showed a slight, non-significant increase in age-standardized melanoma incidence in men (AAPC 0.9%) and stable rates in women (AAPC −0.1%) in Serbia from 2008 to 2022. The rise in diagnosed men largely reflects an aging population rather than higher individual risk. Age-specific analysis confirmed a significant increase in men aged 70–74 (~3% annually), consistent with the known higher melanoma risk in older age.

This finding supports an interpretation that the total growth of crude melanoma rates mostly reflects demographic factors, whereas the changes in the actual risk rates according to the age groups are relatively limited [19]. Additionally, similar patterns have been reported in other European countries with the distinctly pronounced process of population ageing [7]. Such findings highlight the need to consider demographic factors when interpreting temporal trends in malignant diseases.

When comparing our study findings with data obtained from the literature, we note that the rise in melanoma incidence in Serbia was considerably less persistent than in the majority of Western countries. Overall, the incidence of melanoma continues to rise in the majority of fair-skinned populations more rapidly than any other carcinoma [20]. Continuous exposure to ultraviolet (UV) radiation, population ageing, as well as an increased diagnostics capacity—paired with an improved registration process—are possible causes of such a trend [21]. In the past few decades, a continuous rise in melanoma incidence has been reported throughout Europe, specifically in Eastern and Southern regions of the continent, with the most pronounced increases observed in the older age groups (for instance, the AAPC was ~4.0% for males and ~3.0% for females in the period of 1995–2012) [7,22,23]. In the countries of Northern Europe, considering the previously reported high incidence level, a stabilization trend or slight decrease in the incidence rates is observed in younger age groups, whereas older populations still show an increasing trend [3]. In the study conducted by authors from the Great Britain, the incidence of melanoma in young individuals was observed to have remained stagnant in the past few decades, whereas the same incidence was reported to have risen continually in the age groups of those older than 35 years, especially in men (for instance, the AAPC was ~10.4% for older age groups, and ~25.7% for those aged 65 and older) [5]. Such patterns indicate that demographic effects often cause upward shifts in the total (crude) rates, whereas the trends in standardized rates, after removing influences of age structure, may be milder, more stable or even without statistical significance.

Apart from demographic changes, a rise in the incidence of melanoma in Europe is often associated with changes observed in the exposure to ultraviolet (UV) radiation [4,5]. For instance, Garbe et al. [19] reported a persistent rise in melanoma incidence in Germany for the past few decades (the standardized rates increased by approximately 55% from 1999 to 2012), whereas its rise in Serbia was significantly more moderate (for instance, 30% of a relative increase in the ACP values was reported in men during the period of 2008–2022). This indicates that, unlike Germany, where damaging effects of past overexposure of the previous generations to the sun are being manifested now, such a cumulative effect in Serbia has not been achieved yet. However, the fact that only a mild rise was observed in men suggests that Serbia could be at an early phase of a similar trend—for instance, younger male generations who used to follow the skin-tanning and recreational sunbathing trends are now in their middle or older ages, which may subsequently lead to a further increase in incidence rates in the forthcoming years. On the other hand, stability or an insignificant increase in the incidence rates among females in Serbia represents another rather interesting finding. Historically, young Caucasian women had a high melanoma risk due to tanning habits, but recent data show stable or declining rates in this group, likely from behavioral changes and prevention efforts [19]. Furthermore, it is also possible that various campaigns representing the damaging effects of exposure to UV radiation have had a positive impact on younger generations—which is why women tend to take more care of their skin protection and have their skin exams earlier, which contributes to establishing earlier diagnoses of premalignant lesions. All the above-mentioned factors could explain a relatively stagnant trend in the incidence rates of women in our study. The overall trend in melanoma mortality in the Republic of Serbia showed no statistically significant differences over the observed 15-year period. The standardized mortality rates remained considerably stable. Nevertheless, our more thorough analysis revealed certain sex- and age-related specificities that may indicate epidemiologically relevant changes in the mortality pattern. Women in Serbia have recently shown a more favorable trend—which aligns with the reports submitted by a number of European countries, stating that melanoma-specific mortality rates have begun to decline more rapidly in females than in males [24,25]. This is most clearly shown in our study through the continuous decline in mortality rates in women in the middle age group (40–44 years), alongside an abrupt decline in mortality rates for both sexes in the oldest 75+ age group after the period of 2018–2019. Such findings suggest that favorable advances in the treatment outcomes of melanoma can be observed in Serbia in the latest period, particularly among the female population and within the older age groups, which may be associated with advancements in diagnostics and greater availability of modern therapies. These therapies have been proven to extend the survival rates of patients with advanced melanoma, thereby reducing mortality rates in numerous countries [26,27].

A stabilization and decline trend in melanoma-specific mortality aligns with European trends over the past decade. According to the data obtained from the European Cancer Information System (ECIS), melanoma-specific mortality in the EU countries demonstrates a recent flattening of this trend, with a mild decline observed in the majority of Western European countries [8]. Continuously declining mortality rates were reported in Germany, Denmark, the Netherlands and Norway, particularly after the year of 2015, which is attributable to establishing a diagnosis at an earlier stage of disease (through screening programs) and applying new effective therapies [3]. In neighboring Hungary, a significant downward trend in the melanoma-specific mortality rates (by 16.55%) was reported between 2011 and 2019, with the survival rates being significantly improved simultaneously [28,29]. The authors attribute such a positive change to intensive public awareness campaigns aimed at detecting melanoma early, increasing the scope of skin exams (screenings), along with greater availability of modern therapies in that particular period. However, such progress is not equal across the whole of Europe. Numerous Eastern and Southern European countries keep facing less favorable trends, partially due to the later initiation of organized prevention programs and limited availability of innovative medicines [30]. A study that conducted an analysis of the specific melanoma mortality trends, covering 28 European countries in the period from 1960 to 2020, indicated a rise in the mortality rates in both sexes in all age groups in Slovenia and Slovakia, whereas in Croatia, the same rise was reported in the male population, whereas a reduction in the mortality rates was observed in the younger female age groups [24]. Despite certain improvements in the above-mentioned trend that were more than encouraging, the countries of Southeastern Europe are still facing the consequences of late detection, insufficient healthcare funding and delays in the administration of new therapies, leading to the significantly lower survival rates—5-year relative survival rates for melanoma cancer surpass 80% in the majority of Western European countries on average, whereas in some of the countries of Eastern Europe, they are approximately 50% [4]. Serbia fits into this framework due to numerous factors: from a historical perspective, we have slightly lower incidence rates of melanoma, but relatively higher mortality rates compared to wealthier countries, which indicates poorer early detection and inadequate treatment modalities. According to the published studies, the countries of Southeastern Europe have lower incidence rates and higher mortality rates of melanoma compared to Northwestern Europe, which was observed in Serbia as well [31]. Differences in the quality and scope of cancer registry data, along with access to healthcare specialists and modern treatment options, contribute to such disparities. Therefore, this particular research is of paramount significance for public health, as it provides a strong foundation for further monitoring and planning of control mechanisms for cutaneous melanoma in our country.

## 5. Conclusions

The incidence of melanoma in the Republic of Serbia was relatively stable in the period from 2008 to 2022, with a slight increase predominantly limited to the older male population, whereas it remained stagnant in women. Simultaneously, there have not been any significant changes reported in melanoma mortality rates, with clear indications that disease survival rates are gradually being improved due to advances in cancer treatment and early disease detection, since the age-standardized mortality rates have been observed to decline in women in particular.

These findings are encouraging, as Serbia has not yet seen the exponential rise in melanoma incidence observed in other Western countries. However, to prevent future increases due to changing lifestyles, it is crucial to strengthen prevention and early detection strategies. This includes public campaigns promoting sun protection, regulating tanning bed use, organizing skin cancer screenings, educating primary care physicians to identify suspicious lesions, and advising citizens to monitor new or changing moles. Given population ageing and projected melanoma burden growth, sustained investment in prevention, early diagnosis, and monitoring remains a priority to guide future control strategies in Serbia.

## Figures and Tables

**Table 1:** Incidence and mortality of cutaneous melanoma in Serbia, 2008–2022.

Year	Incidence						Mortality						Cancer Survival and Healthcare Quality	
	Men			Women			Men			Women			Men	Women
	N	CR	ASIR	N	CR	ASIR	N	CR	ASMR	N	CR	ASMR	MIR	MIR
2008	271	7.6	4.8	290	7.7	4.9	160	4.5	2.6	98	2.6	1.4	0.54	0.29
2009	320	9	5.5	313	8.3	4.9	151	4.2	2.5	108	2.9	1.4	0.45	0.29
2010	346	9.8	6	310	8.3	4.9	150	4.2	2.7	103	2.8	1.6	0.45	0.33
2011	355	10.1	6.2	338	9.1	5.5	145	4.1	2.7	87	2.3	1.4	0.44	0.25
2012	335	9.6	5.3	312	8.4	4.9	149	4.3	2.6	108	2.9	1.7	0.49	0.35
2013	281	8.1	4.5	266	7.2	3.9	153	4.4	2.6	94	2.6	1.5	0.58	0.38
2014	302	8.7	4.8	296	8.1	4.3	147	4.2	2.5	114	3.1	1.7	0.52	0.40
2015	332	9.6	5.6	302	8.3	4.8	190	5.5	3.4	110	3	1.6	0.61	0.33
2016	372	10.8	6.1	333	9.2	5	159	4.6	2.8	112	3.1	1.7	0.46	0.34
2017	322	9.4	5.3	277	7.7	4.3	166	4.9	2.9	108	3	1.6	0.55	0.37
2018	389	11.4	6.5	320	8.9	5.1	155	4.6	2.7	107	3	1.6	0.42	0.31
2019	364	10.8	6.2	286	8	4.6	175	5.2	3.1	108	3	1.6	0.50	0.35
2020	305	9.1	5.1	265	7.5	4	117	3.5	2.1	85	2.4	1.3	0.41	0.33
2021	370	11.1	5.7	305	8.7	4.7	165	5	2.3	102	2.9	1.3	0.40	0.28
2022	376	11.6	6.2	347	10.2	5.8	153	4.7	2.2	103	3	1.3	0.35	0.22
	5040	9.8	5.6	4560	8.4	4.8	2335	4.5	2.6	1547	2.8	1.5	0.46	0.31

N—number of cases, CR—crude rate, ASIR—age-standardized incidence rate, ASMR—age-standardized mortality rate, MIR—mortality-to-incidence ratio.

**Table 2:** Distribution of cutaneous melanoma incidence and mortality by age groups in Serbia, 2008–2022.

Age Groups	Incidence			Mortality		
	Men	Women	Total	Men	Women	Total
	N (%)	N (%)	N (%)	N (%)	N (%)	N (%)
0–14	0 (0.0%)	0 (0.0%)	0 (0.0%)	0 (0.0%)	0 (0.0%)	0 (0.0%)
15–19	5 (0.1%)	14 (0.3%)	19 (0.2%)	1 (0.0%)	4 (0.3%)	5 (0.1%)
20–24	35 (0.7%)	49 (1.1%)	84 (0.9%)	7 (0.3%)	1 (0.1%)	8 (0.2%)
25–29	54 (1.1%)	83 (1.8%)	137 (1.4%)	11 (0.5%)	12 (0.8%)	23 (0.6%)
30–34	143 (2.8%)	167 (3.7%)	310 (3.2%)	34 (1.5%)	28 (1.8%)	62 (1.6%)
35–39	178 (3.5%)	219 (4.8%)	397 (4.1%)	56 (2.4%)	35 (2.3%)	91 (2.3%)
40–44	250 (5.0%)	287 (6.3%)	537 (5.6%)	71 (3.0%)	67 (4.3%)	138 (3.6%)
45–49	298 (5.9%)	336 (7.4%)	634 (6.6%)	120 (5.1%)	85 (5.5%)	205 (5.3%)
50–54	433 (8.6%)	379 (8.3%)	812 (8.5%)	163 (7.0%)	97 (6.3%)	260 (6.7%)
55–59	612 (12.1%)	515 (11.3%)	1127 (11.7%)	262 (11.2%)	149 (9.6%)	411 (10.6%)
60–64	689 (13.7%)	547 (12.0%)	1236 (12.9%)	292 (12.5%)	146 (9.4%)	438 (11.3%)
65–69	722 (14.3%)	548 (12.0%)	1270 (13.2%)	332 (14.2%)	197 (12.7%)	529 (13.6%)
70–74	680 (13.5%)	559 (12.3%)	1239 (12.9%)	342 (14.6%)	217 (14.0%)	559 (14.4%)
75+	941 (18.7%)	857 (18.8%)	1798 (18.7%)	644 (27.6%)	509 (32.9%)	1153 (29.7%)
Total	5040 (100%)	4560 (100%)	9600 (100%)	2335 (100%)	1547 (100%)	3882 (100%)

**Table 3:** Joinpoint regression analysis of cutaneous melanoma incidence by age groups in Serbia, 2000–2022.

Age Groups	Cohort	Segment	Period	APC/AAPC (95% CI)	*p*
35–39	Men	1	2008–2022	1.5 (−1.4–4.4)	0.295
	Women	1	2008–2022	1.9 (−1.2–5.0)	0.214
40–44	Men	1	2008–2022	1.3 (−2.6–5.3)	0.496
	Women	1	2008–2022	1.8 (−5.3–9.5)	0.600
45–49	Men	1	2008–2022	−0.1 (−2.7–2.5)	0.908
	Women	1	2008–2022	1.8 (−1.5–5.3)	0.266
50–54	Men	1	2008–2022	2.4 (−0.1–4.9)	0.060
	Women	1	2008–2022	0.4 (−2.8–3.7)	0.788
55–59	Men	1	2008–2022	−0.7 (−2.7–1.3)	0.450
	Women	1	2008–2022	−0.1 (−2.0–1.8)	0.912
60–64	Men	1	2008–2022	0.0 (−2.5–2.6)	0.996
	Women	1	2008–2022	−1.5 (−4.0–1.1)	0.224
65–69	Men	1	2008–2022	0.8 (−1.2–3.0)	0.407
	Women	1	2008–2022	1.9 (−1.3–5.1)	0.225
70–74	Men	1	2008–2022	3.1 (0.8–5.4)	**0.011**
	Women	1	2008–2022	0.8 (−0.7–2.2)	0.265
75+	Men	1	2008–2022	1.0 (−1.4–3.5)	0.374
	Women	1	2008–2022	−1.4 (−3.6–0.9)	0.203

APC—Annual Percent Change, AAPC—Average Annual Percent Change.

**Table 4:** Joinpoint regression analysis of cutaneous melanoma mortality by age group in Serbia, 2000–2022.

	Cohort	Segment	Period	APC (95% CI)	*p*	AAPC (95% CI)	*p*
35–39	Men	1	2008–2022	−3.1 (−10.0–4.4)	0.382	— *	—
	Women	1	2008–2022	−0.9 (−8.9–7.7)	0.811	—	—
40–44	Men	1	2008–2022	−3.1 (−11.3–5.9)	0.458	—	—
	Women	1	2008–2022	−7.0 (−12.3–−1.5)	**0.017**	—	—
45–49	Men	1	2008–2022	0.4 (−3.4–4.4)	0.814	—	—
	Women	1	2008–2022	4.0 (−2.6–11.0)	0.218	—	—
50–54	Men	1	2008–2022	−2.2 (−5.4–1.2)	0.181	—	—
	Women	1	2008–2022	−2.4 (−9.2–5.0)	0.486	—	—
55–59	Men	1	2008–2022	−4.0 (−7.9–0.1)	0.054	—	—
	Women	1	2008–2022	−2.7 (−21.0–19.7)	0.778	—	—
60–64	Men	1	2008–2022	−0.8 (−3.1–1.5)	0.442	—	—
	Women	1	2008–2022	−1.7 (−6.1–2.9)	0.428	—	—
65–69	Men	1	2008–2022	−1.3 (−4.0–1.4)	0.311	−1.3 (−4.0–1.4)	0.311
	Women	1	2008–2010	115.6 (−5.1–389.9)	0.064	8.0 (−3.2–20.5)	0.168
	Women	2	2010–2022	−3.8 (−8.3–1.0)	0.110		
70–74	Men	1	2008–2022	1.8 (−0.6–4.2)	0.123	—	—
	Women	1	2008–2022	1.4 (−3.1–6.0)	0.527	—	—
75+	Men	1	2008–2010	45.9 (−12.1–142.1)	0.121	3.1 (−4.6–11.5)	0.442
	Men	2	2010–2018	5.1 (−1.8–12.4)	0.127		
	Men	3	2018–2022	−16.6 (−28.9–−2.1)	**0.032**		
	Women	1	2008–2019	9.1 (4.2–14.2)	**0.002**	−2.9 (−9.6–4.3)	0.421
	Women	2	2019–2022	−36.5 (−54.8–−10.9)	**0.014**		

APC—Annual Percent Change, AAPC—Average Annual Percent Change; * AAPC = APC.

## Data Availability

All data is contained within this article.
